# A systematic literature review and meta-analysis of the incidence of serious or severe hypersensitivity reactions after administration of ferric derisomaltose or ferric carboxymaltose

**DOI:** 10.1007/s11096-023-01548-2

**Published:** 2023-04-03

**Authors:** Nicholas A. Kennedy, Maureen M. Achebe, Patrick Biggar, Johannes Pöhlmann, Richard F. Pollock

**Affiliations:** 1 Department of Gastroenterology, Royal Devon and Exeter NHS Foundation Trust, Exeter, UK; 2 Brigham and Women’s Hospital, Dana Farber Cancer Institute, Harvard Medical School, Boston, USA; 3 Department of Nephrology, Klinikum Coburg GmbH, Coburg, Germany; 4 Covalence Research Ltd, Rivers Lodge, West Common, Harpenden, AL5 2JD UK

**Keywords:** Administration, intravenous, Ferric carboxymaltose, Ferric derisomaltose, Hypersensitivity, Iron, Iron deficiency anemia, Iron isomaltoside

## Abstract

**Background:**

Intravenous iron is the preferred treatment for patients with iron deficiency anemia in a variety of clinical situations. Although uncommon, administration of modern IV iron formulations can result in hypersensitivity reactions (HSRs) and, rarely, anaphylactic or anaphylactoid reactions.

**Aim:**

The objective of the present study was to systematically review the literature to identify and analyze data on the incidence of HSRs after administration of ferric derisomaltose (FDI) or ferric carboxymaltose (FCM).

**Method:**

A prospectively-registered systematic literature review was conducted to identify prospective randomized controlled trials comparing FDI and FCM with other intravenous iron formulations or oral iron. Searches were conducted in PubMed (including MEDLINE), EMBASE, and the Cochrane Library in November 2020. The relative incidence of serious or severe HSRs occurring on the day or day after dosing of intravenous iron, recorded under the standardized Medical Dictionary for Regulatory Activities query for anaphylactic reaction.

**Results:**

Data were obtained from seven randomized controlled trials of FCM (N = 2683) and ten of FDI (N = 3474) enrolling 10,467 patients in total. The number of patients experiencing any serious or severe HSR event was 29/2683 (1.08%) with FCM versus 5/3474 with FDI (0.14%). Bayesian inference of proportions showed the event rates to be significantly lower with FDI relative to FCM.

**Conclusion:**

HSR events were uncommon with both intravenous iron formulations; however, the present study showed a significantly lower incidence of HSRs with FDI relative to FCM. Further large-scale, head-to-head trials of the iron formulations would be required to confirm this finding.

**Supplementary Information:**

The online version contains supplementary material available at 10.1007/s11096-023-01548-2.

## Impact statements

- Incidence of serious or severe hypersensitivity reactions was low with both ferric derisomaltose and ferric carboxymaltose, but the analysis showed a significantly lower incidence of hypersensitivity reactions with ferric derisomaltose versus ferric carboxymaltose.
- The rarity of the events would mean that further, large-scale, head-to-head trials of the iron formulations would be required to confirm this finding.

## Introduction

Intravenous (IV) iron is well established as a successful treatment for patients with iron deficiency (ID) and iron deficiency anaemia (IDA) associated with a diverse range of etiologies, including renal, gastroenterological, gynaecological, and oncological [1–4]. In recent years, the use IV iron treatment has expanded further, most notably in patients with heart failure [5–9] and in patients undergoing surgery where exogenous iron plays a key role in perioperative blood management programs [10–13].

The earliest IV iron formulations were associated with unacceptably high rates of adverse drug reactions caused by ‘labile’ or ‘rapid’ iron release [14, 15]. While the exact mechanism driving hypersensitivity reactions (HSRs) after infusion of IV iron is not currently understood, one hypothesis is that, in addition to reactions due to small amounts of labile iron, the iron-carbohydrate complexes can result in a complement-mediated pseudoallergic reaction (CARPA) [16, 17]. Modern IV iron formulations such as ferric derisomaltose (Monofer®/Monoferric®; Pharmacosmos A/S, Holbaek, Denmark; FDI; formerly known as iron isomaltoside) and ferric carboxymaltose (Ferinject®/Injectafer®; Vifor France, Paris, France; FCM) can be administered rapidly in high doses, differentiating them from other formulations such as iron sucrose and iron gluconate. The rapid, high-dose administration is facilitated by the small amounts of labile iron in both formulations compared to, e.g. iron sucrose [18].

The safety and efficacy of modern IV iron formulations have been characterized in large-scale systematic literature reviews and meta-analyses. A 2015 meta-analysis of 103 trials showed no increase in the incidence of severe adverse events in patients treated with IV iron (n = 10,390) relative to the control groups, including no iron, placebo, oral, or intramuscular iron (n = 8863) [19]. Other meta-analyses have been conducted in patients with specific etiologies of IDA, including chronic kidney disease (CKD) and IDA during pregnancy, with similar findings regarding safety [20, 21]. In patients with IDA during pregnancy, IV iron resulted in fewer medication reactions than oral iron (relative risk [RR] 0.34; 95% confidence interval [CI] 0.20–0.57), while the risk of serious adverse events was not significantly different between IV iron and oral iron in patients with CKD (RR 1.06, 95% CI 0.88–1.28), nor was the risk of infection (RR 1.31, 95% CI 0.89–1.92).

Studies have also attempted to indirectly compare the relative incidence of HSRs after administration of different IV irons using databases of spontaneous reporting of adverse events. Such studies are inherently limited, however, with underreporting and differential reporting of spontaneous adverse reactions potentially resulting in biased estimates of the relative incidence of HSRs [22–24]. Furthermore, such studies rely on distinct sources of data, such as market share or sales data, to inform total exposure to each IV iron formulation. This use of different data sources for the numerator and denominator is fraught with challenges which, when combined with the spontaneous nature of the event reporting, is unlikely to accurately estimate the relative incidence of HSRs. Given the shortfalls of analyses based on adverse events and pharmacovigilance data, estimates of the relative incidence of HSRs with different IV iron formulations should be conducted using data from studies with fewer intrinsic sources of bias, while simultaneously ensuring that the definition of HSRs and timing of events relative to the IV iron infusion are standardized.

Considering recent RCTs of IV irons that include serious or severe HSR as an endpoint, a systematic review with meta-analysis is needed to demonstrate any potential difference in the rate of HSRs between different IV iron preparations. Further to this requirement, the rigorous classification of serious or severe HSRs is critical to improve comparisons of the safety of different IV irons. One such approach is available in the form of standardized Medical Dictionary for Regulatory Activities (MedDRA) queries (SMQs) [25]. SMQs are validated, predetermined sets of MedDRA terms, grouped together after extensive review, testing, analysis, and expert discussion (www.meddra.org). Using the Anaphylactic Reaction SMQ to evaluate serious or severe hypersensitivity is in line with the approach used by the Center for Drug Evaluation and Research (CDER; a division of the US Food and Drug Administration [FDA]) when they evaluated FCM for a New Drug Application in the US in 2013 [26].

## Aim

The objective of the present study was to systematically review the literature to identify and analyze data on the incidence of hypersensitivity reactions (HSRs) after administration of ferric derisomaltose (FDI) or ferric carboxymaltose (FCM).

## Method

### Literature search strategy

The literature search protocol was prospectively registered in PROSPERO, the international prospective register of systematic reviews, with ID CRD42020215727 [27]. Literature searches were developed using free-text terms and Medical Subject Heading (MeSH) terms. Separate searches were conducted to identify studies of FDI versus any oral or IV iron formulation, and FCM versus any oral or IV iron formulation (including FDI; Supplementary Tables 1 and 2). Studies were retrieved from PubMed (including MEDLINE), EMBASE, and the Cochrane Library and imported into Sourcerer (Covalence Research Ltd, Harpenden, UK). Websites of regulatory authorities were also searched, including the European Medicines Agency (EMA), the FDA, and the Medicines and Healthcare products Regulatory Agency. After retrieval, duplicates were automatically removed, leaving two corpora of unique publications (FDI and FCM). Two reviewers (RFP and JP) independently conducted first-round title and abstract screening against the pre-specified exclusion criteria (Table 1). Inter-reviewer discrepancies after title and abstract screening were resolved by discussion, and the full-text articles of the included studies were obtained and screened independently by the same two reviewers against the same exclusion criteria (RFP and JP).

**Table 1:** Exclusion criteria and example reasons for exclusion from the systematic literature review

Reason for exclusion	Example reasons for exclusion
Not a randomized controlled trial	Single arm study, observational study, or retrospective database analysis
Not in the target population	Not in an adult (≥ 18 years) population with IDA
Not investigating the target intervention	Does not include iron isomaltoside or ferric carboxymaltose
Not comparing the intervention with a relevant comparator	Compared with placebo, erythropoietin, or red blood cell transfusion
Not reporting endpoint of interest	Not reporting serious or severe hypersensitivity reactions (HSRs) in line with the standardized MedDRA query for anaphylactic reaction
Not in English	Original article not written in English or with an English translation available

Studies eligible for inclusion were limited to prospectively-designed, active comparator, randomized controlled trials (RCTs) of either FCM or FDI compared with any other IV or oral iron formulation for the treatment of patients with IDA. Extension studies were excluded.

In line with the study protocol, where trials otherwise meeting the review inclusion/exclusion criteria were identified that did not report the specific hypersensitivity endpoint, corresponding authors were contacted with a view to obtaining data on file. The correspondence included a link to the PROSPERO protocol, which in turn included details of the proposed research methodology, source of funding for the research, and plan for dissemination of the findings.

### Data extraction

We searched the included trials for the proportions of patients experiencing a serious or severe hypersensitivity reaction on the day or day after dosing of IV iron, categorized using the SMQ for anaphylactic reaction, and reported under SMQ groups A–D (Supplementary Table 3). All studies were assessed for bias using the Cochrane risk-of-bias tool version 2 (RoB2) for randomized trials [28].

### Data analysis

The *a priori* data analysis plan was informed in part by a 2020 study that focused specifically on evaluating and comparing different methods of indirect comparison of the incidence of HSRs after administration of IV iron [29]. The analysis plan was expanded for the present study on the grounds that additional data may be made available from contacts with the study corresponding authors. Three analysis scenarios were outlined, in which the searches yielded clinically and statistically homogeneous data with or without closed loops in the evidence network, and in which the searches yielded either clinically and statistically heterogeneous data or data that precluded the derivation of appropriate arm-level weightings. Ultimately the latter scenario was employed as not all data could be obtained in a format that enabled study arm-level weightings to be calculated. Two distinct statistical methods proposed in the latter scenario are detailed below.

#### Bayesian analysis

We performed analyses of the naively-pooled data on the incidence of serious or severe HSRs on the day or day after administration of each of the IV iron formulations in SMQ groups and group combinations using Bayesian inference of proportions. To capture the uncertainty around the relative incidence with each of the IV iron formulations, we employed flat, uninformative priors, based on beta distributions parameterized with shape and scale parameters set to 1.

The Bayesian inference of proportions analyses were conducted using Just Another Gibbs Sampler (JAGS) using the RJAGS package [30]. Gelman-Rubin statistics and Gelman plots were used to establish whether the chains had converged [31]. The number of tuning and burn-in steps were set to 500 and 1000, respectively, at which point results were stable. Plots of the posterior distributions were generated, including regions of practical equivalent (ROPEs), which covered an odds ratio range of 0.9–1.1. Uncertainty around the mean odds ratio was summarized using 95% highest posterior density intervals (HDI), corresponding to the interval containing 95% of the posterior distribution of odds ratios.

#### Frequentist naïve pooling

We pooled data on the incidence of all serious or severe HSRs occurring on the day or day after administration of IV iron across all of the study arms for FDI and FCM, split by SMQ group. Odds ratios were then derived directly from the pooled numbers of patients experiencing events and the number of patients treated, with the significance of the results estimated using Fisher’s exact tests, and confidence intervals around the mean odds ratios derived using the Clopper-Pearson methodology [32].

## Results

**Fig. 1:**
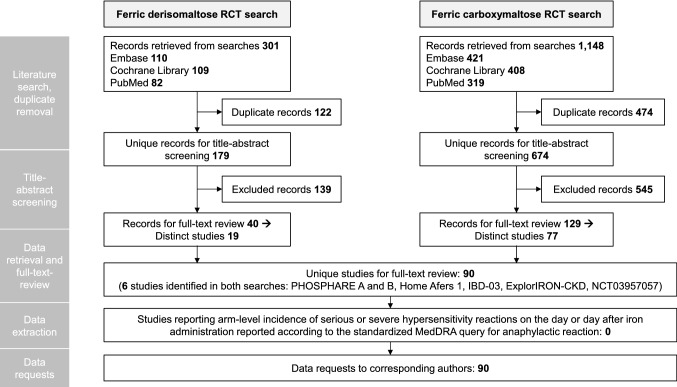
PRISMA flow diagram showing the two searches to identify randomized controlled trials comparing ferric derisomaltose and ferric carboxymaltose with any other intravenous or oral iron formulation

Literature searches were conducted on November 4, 2020. The FDI searches retrieved 301 records, of which 122 were duplicates across the databases, leaving 179 unique records for screening. The FCM searches retrieved 1,148 records, of which 474 were duplicates, leaving 674 unique records for screening. After title and abstract screening by two independent reviewers and resolution of inter-reviewer disagreements (N = 26 across both searches), 545 FCM records and 139 FDI records were definitively excluded. Of the remaining studies, 129 FCM records (corresponding to 77 unique trials) and 40 FDI records (corresponding to 19 unique trials) were included for full-text screening. Six of the 96 total studies across the two searches were head-to-head comparisons of FDI and FCM, leaving records pertaining to 90 unique studies for full-text review (Fig. 1).

**Fig. 2:**
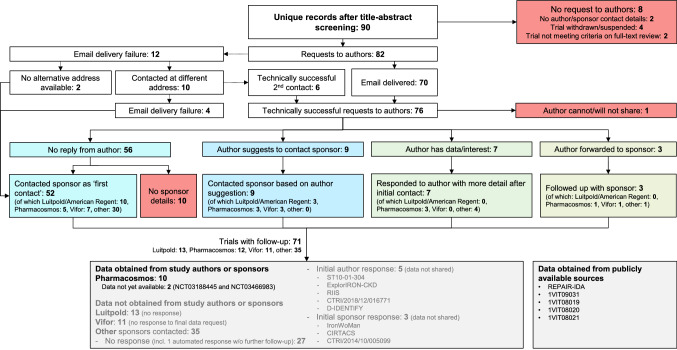
Flow diagram for data requests and incorporation of publicly available data

None of the publications identified reported the incidence of serious or severe hypersensitivity reactions occurring on the day or the day after administration of IV iron using the preferred MedDRA terms falling under the Anaphylactic Reaction SMQ (groups A, B, C, or D). Of the 90 unique studies, four had been withdrawn or suspended, two did not meet the study inclusion criteria on examination of the full-text publication, and two did not include any author or sponsor contact details. Data requests were sent to corresponding authors of the remaining 82 studies describing the study objective (including the PROSPERO protocol), and enquiring as to whether data on file could be made available for analysis (Fig. 2).

As no data were ultimately received from Vifor (the marketing authorization holder for FCM), Luitpold Pharmaceuticals, Inc. or American Regent (the US licensees of FCM), the only source of data for FCM was therefore the 2013 FDA CDER Medical Review of FCM, which presented pooled data on the incidence of serious or severe HSRs across the four SMQ groups from five RCTs: REPAIR-IDA, 1VIT09031, 1VIT08019, 1VIT08020 and 1VIT08021 [33–36]. The REPAIR-IDA and 1VIT09031 studies were designed to assess the cardiovascular safety of the 750 mg dose of FCM, with the former being conducted in a population of patients with non-dialysis dependent CKD, and the latter being conducted in population with a mixture of IDA etiologies [33, 34]. The 1VIT08019, 1VIT08020, and 1VIT08021 studies also investigated the 750 mg dose of FCM [35, 36].

Ultimately, data from 15 RCTs were made available for analysis, seven of which compared FCM with other IV iron formulations or standard medical care, and ten of which compared FDI with other IV iron formulations (including two with FCM) or oral iron [33–45]. The studies included a total of 10,467 patients, of whom 6157 patients who had received either FDI or FCM; 3474 patients treated with FDI and 2683 patients treated with FCM (Table 2). Data for five of the studies of FCM were obtained from the FDA CDER Medical Review of FCM [26]. Across the five RCTs, 2,566 patients were treated with FCM, of whom 28 (1.1%) experienced HSRs across all groups in the SMQ for anaphylactic reactions [26]. Four studies were at low risk of bias, ten had some concerns, and one was classified as being at high risk of bias (Supplementary Fig. 1).

**Table 2:** Patients experiencing serious or severe treatment-emergent adverse event occurring on the day of dosing or the day after dosing that included any term in the standardized MedDRA query (SMQ)

SMQ group	FDI (N = 3474)	FCM (N = 2683)
Any serious or severe adverse reaction (A + B + C + D)	5 (0.1%)	29 (1.1%)
Narrow terms pertaining to hypersensitivity reactions (A)	1 (0.0%)	2 (0.1%)
Broad terms pertaining to respiratory reactions potentially related to hypersensitivity (B)	2 (0.1%)	16 (0.6%)
Broad terms pertaining to skin reactions potentially related to hypersensitivity (C)	1 (0.0%)	3 (0.1%)
Broad terms pertaining to cardiovascular reactions potentially related to hypersensitivity (D)	1 (0.0%)	8 (0.3%)

*FCM* ferric carboxymaltose, *FDI* ferric derisomaltose

Data requests for serious or severe HSR incidence data were ultimately fulfilled for 10 RCTs sponsored by Pharmacosmos A/S via the contacts listed in the respective trial records on clinicaltrials.gov. Data for two RCTs included in the data request were not provided on the grounds that the final study manuscripts had not yet been published [46, 47]. Of the 10 studies for which data were available, four were conducted in patient populations with mixed etiologies of IDA, three in patients with CKD, and one each in patients with inflammatory bowel disease, postpartum hemorrhage, and non-myeloid malignancies receiving chemotherapy.

**Fig. 3:**
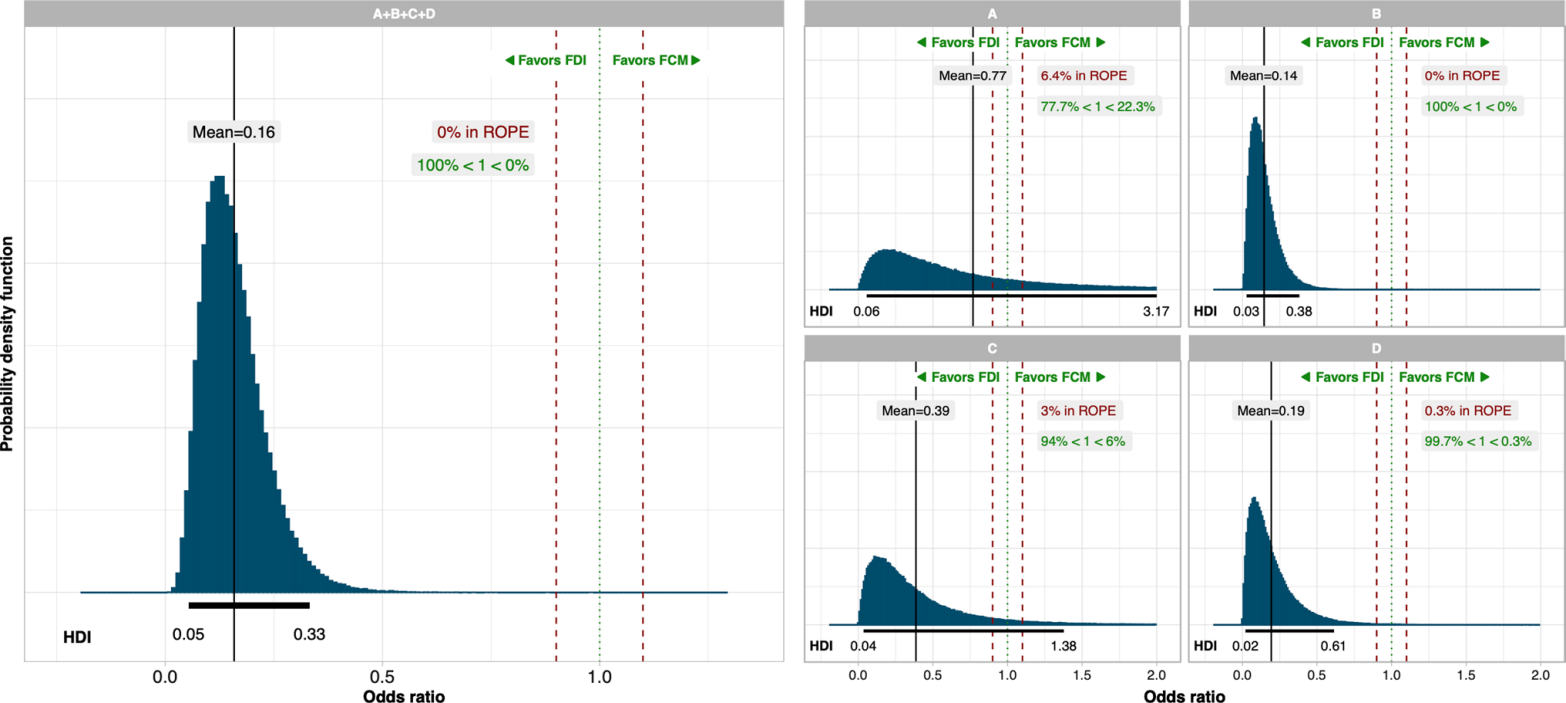
Posterior distributions from the Bayesian inference of proportions of the odds of serious or severe hypersensitivity reactions occurring on the day or day after administration of intravenous iron for FDI versus FCM. Green dotted line indicates parity (odds ratio of 1); red dashed lines indicate the region of practical equivalence (ROPE). *A* Narrow terms pertaining to hypersensitivity reactions, *B* broad terms pertaining to respiratory reactions potentially related to hypersensitivity, *C* broad terms pertaining to skin reactions potentially related to hypersensitivity, *D* broad terms pertaining to cardiovascular reactions potentially related to hypersensitivity, *FCM* ferric carboxymaltose, *FDI* ferric derisomaltose, *HDI* highest posterior density interval, *ROPE* region of practical equivalence

Bayesian analysis of proportions showed that FDI would result in a significant reduction in the incidence of HSRs across SMQ groups A + B + C + D relative to FCM, with a mean odds ratio of 0.16 (95% HDI: 0.05–0.33; Fig. 3) and 0% of the posterior distribution falling within the ROPE. The greatest reductions in odds occurred in SMQ groups B and D, with odds ratios of 0.14 and 0.19, respectively, while SMQ group A (comprising narrow terms pertaining to hypersensitivity reactions) showed the smallest reduction in odds and a high degree of uncertainty, with a mean odds ratio of 0.77 and a highest density interval spanning 0.06–3.17.

**Fig. 4:**
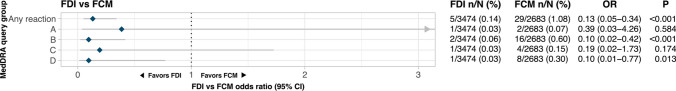
Naïve pooled analysis of the safety of ferric derisomaltose (FDI) versus ferric carboxymaltose (FCM) with odds ratios and confidence intervals of serious or severe hypersensitivity reactions occurring on the day or day after administration of intravenous iron for FDI versus FCM derived using the Clopper Pearson method, and *p* values derived using the Fishers exact test. *A* Narrow terms pertaining to hypersensitivity reactions, *B* broad terms pertaining to respiratory reactions potentially related to hypersensitivity, *C* broad terms pertaining to skin reactions potentially related to hypersensitivity, *D* broad terms pertaining to cardiovascular reactions potentially related to hypersensitivity, *FCM* ferric carboxymaltose, *FDI* ferric derisomaltose, *MedDRA* Medical Dictionary for Regulatory Activities, *OR* odds ratio

In the naïve pooling approach, with odds ratios derived directly from the event counts, binomial confidence intervals derived using the Clopper Pearson methodology, and *p* values derived using Fishers exact test, the odds of experiencing any serious or severe hypersensitivity reaction with FDI were 87% lower than with FCM (odds ratio 0.13, 95% confidence interval 0.05–0.34, *p* < 0.001; Fig. 4).

## Discussion

This meta-analysis evaluated prospectively collected data from 15 head-to-head RCTs including 10,467 patients with IDA treated with exogenous iron. The Bayesian inference of proportions applied across the pooled data from all trials of FDI versus FCM showed a substantial reduction in the incidence of serious or severe HSRs on the day or day after administration with FDI versus FCM. The key strengths of the analysis lie in the well-specified definition of HSRs in line with that employed by the FDA, and the exclusive use of data from prospective, active comparator, RCTs.

The analysis has some limitations that should be considered when interpreting the findings. While best efforts were made to obtain data from the manufacturer and US licensee of FCM (Vifor Pharma Group and American Regent, respectively), no data were ultimately forthcoming from either party, which restricted the analysis, necessitating the use of data from the CDER report that had already been pooled across multiple trials. Another important limitation was the inability to conduct a more conventional network meta-analysis (NMA) based on the data, despite having limited head-to-head evidence comparing FDI with FCM, FCM with iron sucrose (IS), and FDI with IS. A full NMA was precluded on the grounds that HSR rates across all three IV iron formulations are extremely low and the only two head-to-head trials of FCM and FDI for which HSR incidence data were available enrolled a total of 245 patients, in which only 1 HSR occurred in the FCM arm [45]. The single event in SMQ group B allowed an odds ratio to be calculated for FDI versus FCM, but only by using a correction that results in biasing the estimate towards no difference and overestimating the variance. Utilizing this in an NMA would have resulted in one edge of the network being based on an odds ratio estimate that was intrinsically biased.

Analysis using a Bayesian inference of proportions indicated that FDI would reduce the odds of experiencing serious or severe HSRs in SMQ groups A + B + C + D by 84% relative to FCM (mean odds ratio of 0.16; 95% HDI: 0.05–0.33). The greatest reductions were identified in the analyses of SMQ groups B and D, which reported mean odds ratios of 0.14 and 0.19 respectively, with 95% HDIs that did not cross parity. Our analysis represents, to our knowledge, the most comprehensive effort to synthesize prospectively-gathered evidence on the incidence of serious or severe HSRs in patients treated with modern IV iron formulations conducted to-date, including studies that enrolled over 6000 patients on the two high-dose IV iron formulations. Finally, our analysis is limited to hypersensitivity; other aspects of IV iron safety, particularly risk of infection, hypophosphataemia and cardiovascular events, were not considered.

Despite the limitations inherent in a naively-pooled analysis, the present analysis represents a substantial improvement on approaches relying on combining data from PV and market share data, where there is no guarantee of agreement between the number of patients experiencing HSRs and patient exposure, with market share data additionally not being subject to external scrutiny from peer reviewers, clinicians or marketing authorization holders [13, 14]. The current analysis is not affected by issues of heterogeneous incidence and exposure data, utilizing data exclusively from prospective, active-comparator, RCTs to ensure congruence between exposure and the numbers of patient experiencing events. Furthermore, all serious or severe HSRs were reported using preferred MedDRA terms within the four groups comprising the Anaphylactic Reaction SMQ, ensuring consistency across trials in accordance with validated and pre-specified definitions [48]. Challenges in accurately assessing the safety of IV iron formulations have been documented previously, and we would recommend the use of the Anaphylactic Reaction SMQ groups for the recording and publication of HSR data across future trials of IV iron. Given the rare nature of the serious or severe HSRs with modern IV iron treatments, these data would ideally originate from large-scale head-to-head trials of the modern IV iron formulations, although more data even from smaller trials could still be used to enhance the relative incidence estimates using indirect techniques such as those presented here.

While the present analysis showed a significant reduction in the incidence of HSRs with FDI versus FCM across SMQ groups A + B + C + D, HSRs were uncommon with both iron formulations. In clinical practice, the choice of IV iron formulation should therefore be based on a combination of factors, including the risk of HSRs, risk of other adverse events such as hypophosphatemia, and the maximum allowed single dose, health economic impact, and local availability of each formulation.

## Conclusion

This is, to our knowledge, the first systematic review and meta-analysis investigating the incidence of serious or severe hypersensitivity reactions, using MedDRA preferred terms in the four groups of the SMQ for anaphylactic reaction and so giving a robust comparison of the newer IV irons. We have demonstrated that serious or severe hypersensitivity reactions were uncommon with the newer high dose IV iron formulations and that the incidence of serious or severe HSRs is significantly lower with FDI relative to FCM.

## Supplementary Information

Below is the link to the electronic supplementary material.

Supplementary file1 (DOCX 46 kb)
