# Supplementary material for: A systematic literature review and meta-analysis of the incidence of serious or severe hypersensitivity reactions after administration of ferric derisomaltose or ferric carboxymaltose
Source: Int J Clin Pharm. 2023 Apr 3;45(3):604–12. doi: 10.1007/s11096-023-01548-2 (PMC10250464; doi:10.1007/s11096-023-01548-2)
Supplement: Supplementary file 1 — Supplementary file1 (DOCX 46 kb) [file 11096_2023_1548_MOESM1_ESM.docx]

SUPPLEMENTARY MATERIALS

Supplementary Table 1 Randomized controlled trial search term

| # | Search term |
| --- | --- |
| 1 | randomized controlled trial [pt] |
| 2 | controlled clinical trial [pt] |
| 3 | randomized [tiab] |
| 4 | placebo [tiab] |
| 5 | drug therapy [sh] |
| 6 | randomly [tiab] |
| 7 | trial [tiab] |
| 8 | groups [tiab] |
| 9 | 1 OR 2 OR 3 OR 4 OR 5 OR 6 OR 7 OR 8 |
| 10 | animals [MeSH] NOT humans [MeSH] |
| 11 | 9 NOT 10 |

Supplementary Table 2 Search terms to identify studies on specific intravenous iron formulations

| Iron formulation | Search term |
| --- | --- |
| Iron isomaltoside | Monofer OR Monover OR Monoferric OR 'ferric derisomaltose' OR 'iron isomaltooligosaccharide OR 'iron oligosaccharide' OR iron isomaltopentaoside OR ‘iron isomaltoside’ OR  1345510-43-1.rn. OR AHU547PI9H.af.  AND  inject* OR infus* OR intravenous* OR parenteral* |
| Ferric carboxymaltose | 'ferric carboxymaltose' OR Ferinject OR Injectafer OR  'iron carboxymaltose' OR 9007-72-1.rn. OR 6897GXD6OE.af.  AND  inject* OR infus* OR intravenous* OR parenteral* |

Supplementary Table 3 Preferred MedDRA terms falling in each group of the standardized MedDRA query (SMQ) for anaphylactic reaction

| Group A | Group B | Group C | Group D |
| --- | --- | --- | --- |
| **Narrow terms pertaining to hypersensitivity reactions** | **Broad terms pertaining to respiratory reactions potentially related to hypersensitivity** | **Broad terms pertaining to skin reactions potentially related to hypersensitivity** | **Broad terms pertaining to cardiovascular reactions potentially related to hypersensitivity** |
| - Anaphylactic reaction - Anaphylactoid reaction - Anaphylactic shock - Anaphylactic transfusion reaction - Anaphylactoid shock - Circulatory collapse - First-use syndrome - Kounis syndrome - Shock - Type I hypersensitivity | - Acute respiratory failure - Asthma - Bronchospasm - Cough - Dyspnoea - Respiratory distress - Respiratory failure - Wheezing - Bronchial oedema - Cardiorespiratory distress - Chest discomfort - Choking - Choking sensation - Circumoral oedema - Cyanosis - Hyperventilation - Laryngeal dyspnoea - Laryngeal oedema - Laryngospasm - Laryngotracheal oedema - Mouth swelling - Nasal obstruction - Oedema mouth - Oropharyngeal spasm - Oropharyngeal swelling - Respiratory arrest - Reversible airways obstruction - Sensation of foreign body - Sneezing - Stridor - Swollen tongue - Tachypnoea - Throat tightness - Throat oedema - Tracheal obstruction - Tracheal oedema - Upper airway obstruction | - Angioedema - Flushing - Edema - Pruritus - Rash - Allergic oedema - Erythema - Eye oedema - Eye pruritus - Eye swelling - Eyelid oedema - Face oedema - Generalized erythema - Injection site urticaria - Lip oedema - Lip swelling - Ocular hyperaemia - Oedema - Periorbital oedema - Pruritus allergic - Pruritus generalized - Rash erythematous - Rash generalized - Rash pruritic - Skin swelling - Swelling - Swelling face - Urticaria - Urticaria papular | - Cardiac arrest - Cardio-respiratory arrest - Hypotension - Blood pressure decreased - DBP decreased - SBP decreased - Cardiovascular insufficiency - Diastolic hypotension |

Supplementary Figure 1 Risk of bias assessments of the 15 included randomized controlled trials
